# Increased genetic diversity from colony merging in termites does not improve survival against a fungal pathogen

**DOI:** 10.1038/s41598-020-61278-7

**Published:** 2020-03-06

**Authors:** Carlos M. Aguero, Pierre-André Eyer, Edward L. Vargo

**Affiliations:** Department of Entomology, 2143 TAMU, Texas A&M University, College Station, Texas 77843-2143 USA

**Keywords:** Behavioural ecology, Social evolution, Entomology

## Abstract

In some species of social insects the increased genetic diversity from having multiple breeders in a colony has been shown to improve pathogen resistance. Termite species typically found colonies from single mated pairs and therefore may lack the flexibility to buffer pathogen pressure with increased genetic diversity by varying the initial number of reproductives. However, they can later increase group diversity through colony merging, resulting in a genetically diverse, yet cohesive, workforce. In this study, we investigate whether the increased group diversity from colony fusion benefits social immunity in the subterranean termite *Reticulitermes flavipes*. We confirm previous findings that colonies of *R. flavipes* will readily merge and we show that workers will equally groom nestmates and non-nestmates after merging. Despite this, the survival of these merged colonies was not improved after exposure to a fungal pathogen, but instead leveled to that of the more susceptible or the more resistant colony. Our study brings little support to the hypothesis that colony fusion may improve immunity through an increase of genetic diversity in *R. flavipes*. Instead, we find that following exposure to a lethal pathogen, one colony is heavily influential to the entire group’s survival after merging.

## Introduction

Social insects are among the most abundant and ecologically successful species^1^. Their success is inextricably linked to their division of labor where workers engage in different tasks to benefit one or a few reproductives at the expense of their own reproduction. The low number of reproductives in colonies of most species results in high relatedness among nestmate workers, elevating indirect fitness benefits^2^. Paradoxically, their social life also entails severe costs, as high worker densities, high relatedness, and closed nests strongly increase the chance of pathogen transmission, which would suggest that these species are vulnerable to disease outbreaks^3^. Owing to such pathogenic pressure, social insects have evolved social immunity, whereby individual immune functions and behaviors collectively provide colony-wide disease protection^4–6^. Social immunity includes self/allogrooming^7–14^, nest hygiene^15–22^, removal of diseased individuals^23–26^ and the use of antimicrobial compounds either produced by individuals or from materials incorporated into the nest^27–38^. These diverse immune strategies have undoubtedly reduced the costs of social living, facilitating the success of social insects.

Colony resistance to pathogens is also associated with within-colony genetic diversity, as genetically distinct individuals may vary in their susceptibility to different disease strains^39–45^. Therefore, a mix of distinct genotypes within a colony interferes with genotype x genotype interactions, such that a pathogen able to infect one genotype may fail to transmit to new hosts if it encounters host genotypes that it cannot infect. Consequently, a genetically diverse colony may reduce the overall spread of a pathogen. Although variation in susceptibility may increase the likelihood of an infection, it may also prevent the risk that an outbreak of a single strain of pathogen wipes out all individuals, as a diverse colony will only lose a fraction of its population^41^. In addition, the efficiency of social immunity may increase with within-colony genetic diversity whereby genetically distinct individuals differ in their propensities to detect, survive and respond to different pathogens. Therefore, genetically diverse colonies may be better protected against the threat of a variety of disease agents^3,41,46,47^.

In social Hymenoptera, colonies may increase their level of genetic diversity by the presence of several reproductive queens (i.e., polygyny) and queens mated with several males (i.e., polyandry)^48,49^. Despite these strategies being associated with a reduction of relatedness within colonies (i.e., the indirect fitness of workers), they have evolved several times in social Hymenoptera^50^. One of the main hypotheses suggests that the increased genetic diversity that results from the high number of reproductives in a colony can strengthen their resistance toward pathogens^3,39,46^. Empirical studies have shown improved pathogen resistance from polyandry in bumblebees^51–53^, honeybees^40,42–44,54–57^, and ants^58^, as well as from polygyny in ants^59^. The beneficial effect of genetic diversity on social immunity has been widely examined in social Hymenoptera, but its evidence in termites, the other major group of social insects, is scarce.

Termite colonies share several features with Hymenopteran colonies even though sociality evolved independently in this group^60,61^. However, they differ from social Hymenoptera in that their colonies are founded by a single pair of primary reproductives, the queen and the king. Consequently, as termites have a relatedness initially locked at 0.5, they lack the flexibility to potentially buffer variable pathogen pressure with increased genetic diversity by varying the initial number of reproductives. After the founding stage, though, many termite species are able to merge colonies, which can result in a genetically diverse, yet cohesive, workforce^62^. The factors underlying colony merging are not well understood. Discrimination between nestmates and non-nestmates may play an important role in colony merging. In the subterranean termite, *Reticulitermes flavipes*, merged colonies had strong similarities in their mitochondrial DNA. The close relatedness between merging colonies, associated with a shared maternally inherited factor, may decrease nestmate recognition and favor colony fusion^63^. Potentially, nestmate recognition could also be related to the gut microbiota, as the microbial communities of termite guts are colony-specific^64^. In the species *R. speratus*, colonies can be made to accept foreign workers by experimentally altering the microbial gut communities^65^. In this species, colonies are also more likely to merge if the introduced colony has low proportions of nymphs^66^. Termite nymphs have high resource demands^67^, which makes them energetically expensive for the receiving colony. Thus, colony merging may be influenced by the current status of the colonies involved. The costs and benefits of taking in additional, unrelated workers likely vary across different colonies and species. It is also possible that colony merging could not be a specific behavior that is selected for, but a byproduct of distinct colonies expanding their foraging galleries in the same food source. As they consume more wood and expand their foraging galleries, then those colonies would not be able to remain separate.

Colony merging occurs naturally in *R. flavipes*^68–72^ and this species shows a lack of intercolonial aggression in laboratory assays^73,74^. In this study, we investigated whether the increased group diversity through colony merging benefits social immunity and pathogen resistance in *R. flavipes*. We first confirmed the ability of *R. flavipes* to fuse colonies by investigating merging rate and aggression between pairs of colonies through a behavioral assay. Potentially, merged colonies could preferentially groom their relatives, so we also assessed whether social immunity was symmetrical in artificially merged colonies by comparing the amount of grooming between nestmates and non-nestmates. Finally, we tested the hypothesis that increased group diversity enhances pathogen resistance by determining whether the survival of artificially merged colonies was higher than that of single colonies against an entomopathogenic fungus. Further, we determined if single colonies benefited from specific pairings by comparing the survival of each merged colony pairing to that of its two constituent colonies. Finally, we discuss the ecological and evolutionary factors underlying termite colony merging from the perspective of social immunity.

## Results

### Colony merging

**Figure 1:**
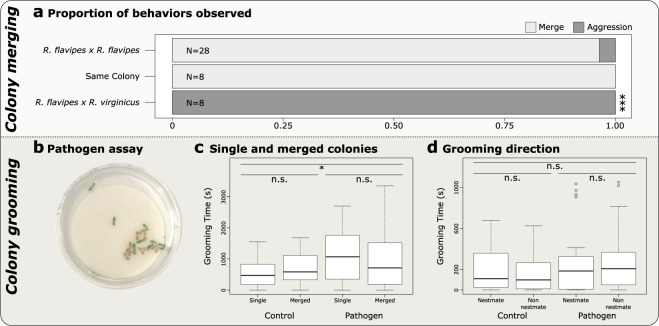
(**a**) The proportion of behaviors observed in each type of pairing in the agonism assay. The asterisks indicates that aggressive behavior was highly associated with interspecific pairings between *R. flavipes* and *R. virginicus* (χ^2^-test, p < 0.0005). (**b**) The pathogen assay arena with examples of mutual grooming between dyed and undyed termites, originally from different colonies. (**c**) Colonies exposed to a pathogen treatment spent more time grooming (Nested ANOVA, p < 0.05), but there was no difference in grooming between single (N = 6) and merged colonies (N = 6) (Nested ANOVA, p = 0.8849). (**d**) There was no difference in the time that termites from merged colonies spent grooming nestmates or non-nestmates (Nested ANOVA, p = 0.90711, N = 6). All analyses were performed in the statistical software R 3.5.0 (https://www.r-project.org/).

Intercolonial aggression and colony merging was determined using a behavioral assay designed to test agonism between groups of termites^75^. We recorded aggressive behaviors exhibited between termites from a colony of *R. flavipes* that were either paired with termites from another *R. flavipes* colony, a group of termites from the same colony, or termites from a colony of the related species, *R. virginicus*. Aggressive behavior was strongly associated with the type of pairing that was tested (χ^2^ = 38.973, *p* < 0.0005; Fig. 1a). Out of the 28 pairings between groups of *R. flavipes* originating from two different colonies, 27 merged without any evidence of aggression. Only one pairing (colonies E and H) resulted in aggression. In the positive control, no aggression was found in pairings between two groups of the same colony. In the negative control, all pairings between colonies of *R. flavipes* and *R. virginicus* displayed aggressive behaviors, showing the ability of *R. flavipes* to exhibit agonistic behaviors while in this experimental setting.

### Grooming behavior

We measured grooming in groups of termite workers that either all originated from the same colony or were artificially merged from two different colonies. In these merged groups, one colony was previously fed a blue dye, so that both colonies could be distinguished from each other (Fig. 1b). These single and merged colony groups were exposed to either a control solution or a solution containing conidia of the fungal pathogen, *Metarhizium robertsii*, at a concentration of 1×10^6^ conidia/mL. Then, we recorded the time spent grooming, as well as whether the grooming was directed towards nestmates or non-nestmates. We observed a significant increase in grooming within termite groups exposed to a pathogen, in both single and artificially merged colonies (*p* < 0.05; Fig. 1c). However, the total duration of grooming did not differ significantly between single and merged colonies, in both control and pathogen groups (*p* = 0.8849; Fig. 1c). In merged colonies, termites did not invest significantly more time in grooming nestmates or non-nestmates, whether they were exposed to a control or pathogen solution (*p* = 0.90711; Fig. 1d).

### Survival of single and merged colonies

**Figure 2:**
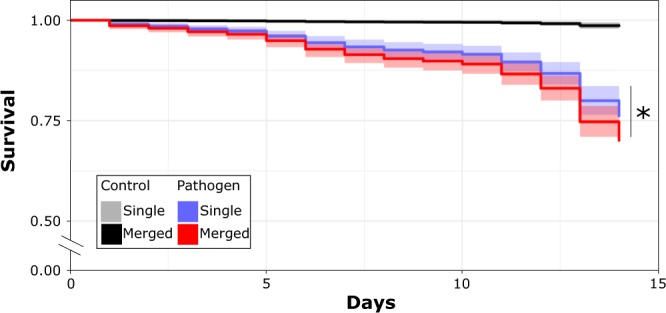
Kaplan-Meier survival distributions of all single (N = 6) and merged colony (N = 6) groups that were exposed to either a control or pathogen solution. Termites exposed to a pathogen had significantly lower survival than termites which received a control solution (p < 0.001). The asterisk indicates that merged colonies were found to have slightly lower survival than single colonies (p < 0.05). All analyses were performed in the statistical software R 3.5.0 (https://www.r-project.org/).

**Figure 3:**
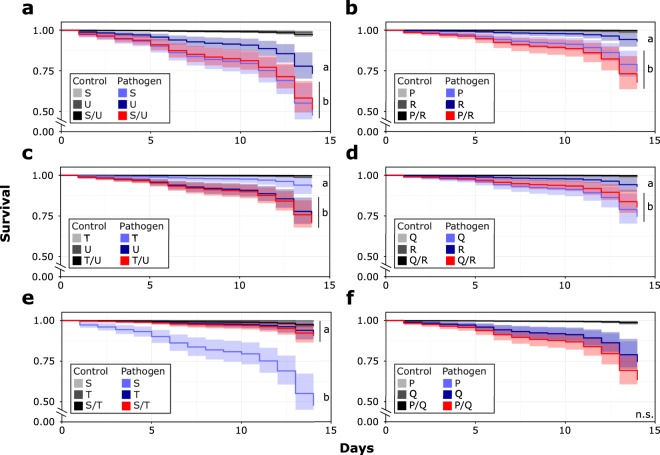
Kaplan-Meier survival distributions of each merged colony pair (Red, N = 1) are plotted with the survival distributions of their corresponding single colonies (Light and dark blue, N = 2). Bolded letters (**a**–**f**) correspond to each of the six groupings of merged and single colonies that were tested. Within each plot, letters denote significant groupings between pathogen treatment groups. The survival distributions of control groups are depicted in the plots, but are not included in groupings. Significance was determined by pairwise comparisons using a log-rank test (p < 0.05; Supplementary Information T1). All analyses were performed in the statistical software R 3.5.0 (https://www.r-project.org/).

Colonies exposed to the pathogen treatment had 20–25% lower survival (*p* < 0.001; Fig. 2). We also found that the survival of merged colonies exposed to a pathogen was slightly lower than the survival of single colonies (*p* < 0.05; Fig. 2). When we examined each specific merged colony pairing, we found that in five out of the six pairing combinations, the two single colonies used to build the artificially merged colony had significantly different survivals. In four of these pairings, the merged colony survival aligned with the survival of the more susceptible of the two single colonies (Fig. 3a–d). In the fifth pairing, the merged colony survival matched the more resistant of the two single colonies (Fig. 3e). In the last pairing, where the survival distributions of the two single colonies were not different from each other, the survival of the merged colony did not differ from either of the two single colonies (Fig. 3f). The results of the pairwise comparisons, including those made between controls are provided in Supplementary Information T1.

## Discussion

We confirmed that colonies of this species readily merge in the lab and showed that workers groom nestmates and non-nestmates equally after merging. These two results are prerequisites for colony merging to test for improved resistance to pathogens. However, the survival of merged colonies was not improved from that of single colonies when challenged with a pathogen. Instead, our results showed that the overall survival of each merged colony was heavily influenced by the survival of the colonies from which it was composed. In most cases, the survival of the merged colony was reduced to that of the more susceptible colony, but in one case the survival of the group was raised to that of the more resistant colony. Our study brings little support to the hypothesis that colony merging may improve immunity through an increase of genetic diversity in *R. flavipes*.

In *R. flavipes*, different colonies have been shown to vary considerably in their ability to resist different strains of pathogens^45^. This finding was supported by our study, as most of the single colonies differed in their survival against the entomopathogenic fungus studied (Fig. 3). However, most studies, including ours, used a single generalist pathogenic agent to challenge colonies. The use of a diverse pathogen cocktail may further test the hypothesis that increased diversity within a colony will provide protection against a broad range of pathogen pressures^41^, while it also better represents natural pressures that colonies encounter. In addition, it would be interesting to test whether the use of generalist or specialist pathogen agents have distinct outcomes on colony survival. Termites can display a vibratory alarm to their nestmates in response to pathogens^76,77^. In *R. flavipes* the strength of this alarm varies between colonies and is positively correlated with the time that nestmates spend grooming, which in turn predicts their survival^78^. Thus, variation in survival may also be influenced by the variation among colonies to detect, and therefore respond or avoid different pathogens. In our study, we ensured the exposure of individuals to the pathogen by flooding the substrate with a pathogen suspension in a small arena. This setup allows the infection of all termites to measure differential survival, but hampers their survival through avoidance. We also showed for the first time that workers equally groom nestmates and non-nestmates after merging (Fig. 1d). One would therefore expect merged colonies to survive at the level of the more resistant colony, as workers with greater detection ability should be able to groom the entire merged colony. However, our finding that survival is lower in merged colonies indicates that the more susceptible colony may determine the level of group susceptibility.

Several mechanisms of social immunity have been examined in termites. Nest structures are typically constructed from fecal material, which can inhibit the growth of harmful microbes^79^. Termite nests have also been found to harbor beneficial Actinobacteria, which are known to possess antimicrobial activity^35,38^. Colonies can maintain hygienic conditions by cannibalizing nestmates that are infected or have recently succumbed to disease^25,80–82^. Additionally, the ‘social transfer’ of disease resistance has been reported in the dampwood termite species, *Zootermopsis angusticollis*, where individuals have improved survival against a fungal pathogen after being grouped with individuals that have survived a challenge with the same pathogen^83^. However, grooming is one of the most effective mechanisms of social immunity that has been studied in termites. Workers kept in isolation have much greater mortality than those kept in groups after exposure to a fungal pathogen^10,13,84^. Termite salivary glands produce antimicrobial peptides which, when applied though grooming, effectively inhibit the growth of fungal pathogens^85–87^. In addition, grooming allows termites to remove fungal spores attached to the cuticle of a nestmate. These spores are swallowed and ultimately end up in the gut of their nestmates, where they are unable to germinate^13,88^. Even once the conidia have penetrated the cuticle, and the infected termite can no longer be saved, nestmates still show a large grooming response^25^. This intense grooming may also ultimately lead to cannibalism to prevent dying individuals from proliferating disease. The unyielding nature of termite grooming behavior may help provide constant protection from disease spreading throughout the colony.

Termite colonies undergo developmental changes throughout their lifespan, which may affect the role that genetic diversity plays in immunity. In many termite species, including *R. flavipes*, genetic diversity may decline over time due to the development of secondary reproductives in the colony (i.e., transition to an extended-family)^62,89^. When the founding king and queen perish, the workers or nymphs of the colony may become secondary kings and queens that engage in repeated inbreeding over time. These secondary reproductives prolong the life of the colony at the expense of reduced genetic diversity within the colony. Thus, extended-family colonies may be more likely to merge in order to restore the level of genetic diversity within colonies. Indeed, naturally merged colonies of *R. flavipes* appear to merge shortly after the death of the founders of one of the constituent colonies^90^. Genetic diversity may not only be important to older colonies that experience inbreeding, but also to those that are still young. In *R. flavipes* and *R. virginicus*, the proportion of mature colonies headed by inbred reproductives is lower than the proportion of inbred pairs found during the nuptial flight suggesting that inbreeding depression negatively affects developing colonies^91^. Colony foundation represents an important threshold for termite survival, as young colonies undergo strong selective pressure^92^. In *Z. angusticollis*, inbreeding between founding kings and queens produces offspring with increased disease susceptibility in comparison to outbred mating^93^. It has also been shown that the effects of pathogen exposure during early colony foundation constrain the reproductive output and overall survival of the future colony in this species^94^. The king and queen play pivotal roles in incipient colonies. Until the workforce is large enough to completely sustain the colony, the king and queen are responsible for protecting themselves and rearing the first brood. Thus, the survival of incipient colonies is largely dependent on the fitness of the founding pair^94–96^.

In social Hymenoptera, strong support for improved disease resistance from increased genetic diversity has been demonstrated in bumblebees^51–53^ and honeybees^40,42–44,54–57^, while similar experiments in ants have produced contrasting results. While several ant species show no relationship between genetic diversity and pathogen resistance^97–99^, there are some that do. In the leaf-cutting ant, *Acromrymex echinatior*, workers from different patrilines vary in their susceptibility to a fungal pathogen, suggesting that a genetically diverse worker force may increase the overall colony survival against a variety of pathogens^58^. In *Formica selysi*, naturally polygynous colonies do not have higher survival than monogynous colonies, but artificially combined workers from different monogynous colonies do show improved disease resistance^59^. While there is some indication that genetic diversity could provide health benefits for both ants and termites, strong evidence is still lacking for its relevance in natural settings.

In *R. flavipes* colonies vary in their ability to detect and respond to disease, they will readily merge, and workers will groom each other equally in merged colonies. Despite this, we do not find evidence that genetic diversity improves pathogen resistance in *R. flavipes*. Instead we observed that after merging, one colony heavily influenced the survival of the group. The factors that determine which colony is more influential to immunity after merging remain unknown. Additionally, there are some caveats to this study that may warrant further investigation. While our method of testing merging rates among colony fragments is sufficient to ensure that our merged colonies would not attack each other during the experiment, termites may still maintain colony boundaries without aggression. A study examining whether colony fragments of *R. flavipes* would share foraging and nesting sites reported merging rates to be as low as 55% in laboratory assays^74^. In our study, we found balanced grooming within the merged colonies, but the interactions between colonies that have fused may be more complex. Potentially, by allowing mixed colonies to acclimate together, the two colonies may have developed a combined colony odor that may preclude any kin recognition in grooming. As with what has been found in ants, the relationship between genetic diversity and immunity in termites may vary among species. Further studies investigating the proximate mechanisms by which changes in the genetic architecture of merged colonies, such as an increase in allelic diversity, heterozygosity, or differential gene expression, would affect colony survival are clearly worth more attention. Investigations into the potential benefits of colony merging in this and other species are needed to determine whether fitness gains derived from increased genetic diversity drive colony merging in termites.

## Methods

### Termite and pathogen collection

We collected groups of termites from 20 colonies of *R. flavipes* and one colony of the related species *R. virginicus*. The colonies were sampled from wood debris found in College Station, Texas from October 2018 to February 2019. Based on previous studies, all collections were made at least 15 m apart to ensure that each colony was unique^90,100,101^. In *R. flavipes*, mate pairing is random during large, synchronous nuptial flights, leading to an absence of isolation-by-distance patterns at short distances, meaning that two geographically close colonies are not genetically more similar than two geographically distant colonies^69,100,102,103^. As a consequence, our sampled colonies likely represent a spectrum of varying levels of relatedness, regardless of their sampling distances. The termites were separated from wood in the lab, assigned an identifying letter (A-T) and maintained in rearing chambers at 85% relative humidity and 27 °C. All termites were used in experiments within 2 weeks of collection. The DNA of one worker per colony was extracted and sequenced at the 16 S mitochondrial locus to confirm the species identification of the colonies sampled (Supplementary Information S1).

A field-collected strain of the fungus *Metarhizium robertsii* was used in the pathogenic treatment for this study. This strain was isolated from soil collected from the Sam Houston National Forest, TX using a mealworm baiting method, then cultured on a medium of potato dextrose agar^104^. The identity of the strain was confirmed through sequencing analysis of the ITS region (Supplementary Information S1). Fungal conidia were collected from the medium and suspended in a 0.1% TWEEN80 solution. This solution was concentrated at 1 × 10^6^ conidia/mL using a hemocytometer (Bulldog Bio, Inc. Portsmouth, NH, USA) and used as a pathogen treatment. The 0.1% TWEEN80 solution without fungal conidia was used as a control. These pathogen and control solutions were used for all immune challenges in this study.

### Colony merging

We performed an agonism assay using the arena described in Chouvenc & Su 2017. Ten workers and one soldier from two different colonies were introduced into opposite sides of a 3×10 cm arena and were allowed to tunnel through sand along a preformed path, until the two groups encountered each other^75^. We tested every combination of eight different *R. flavipes* colonies (colonies A-H; N = 28) and recorded any signs of aggression between colonies (biting, tunnel blocking, casualties, etc.). As controls, we also tested all eight of these colonies against themselves (N = 8), as well as against the colony of *R. virginicus* (N = 8). All pairings were monitored for behavior every 15 minutes for 3 hours after introduction, which was enough time for termites from both sides to connect the tunnels and interact with each other. Pairings were denoted as aggressive if any signs of aggression between the two groups was observed. The two groups were considered to have merged if there were no signs of aggression and workers freely moved between both sides of the assay.

### Grooming behavior

Six colonies (colonies I-N) were used to determine the amount of grooming in the presence of pathogens, as well as to compare the amount of grooming between nestmates and non-nestmates within artificially merged colonies. At the time of collection, a subset of workers from each colony were fed cellulose material containing Nile blue (a fat-soluble stain used to mark termites) so that workers from different colonies could be identified when mixed with another colony (Fig. 1b). Nile blue has been used in a number of studies to mark termites and has not been reported to affect termite behavior^25,83,105,106^. Groups of 20 workers were set up with either 20 workers from the same colony (i.e., single colony), or by combining 10 dyed workers from one colony with 10 undyed workers from another colony to create six merged colonies (**J**xK, Jx**L**, **K**xL, **M**xN, Mx**O** and **N**xO; bolded letters indicate the dyed colony). Groups were isolated in 60 mm petri dishes with moist filter paper and allowed to acclimate for 1 week before exposure. Two identical replicates were performed for all single colony (N = 6) and merged colony (N = 6) groups. Pathogen and control solutions were applied by pipetting 200 μL of solution onto the filter paper in each petri dish. Five-minute videos were recorded for each petri dish 15 minutes after the solution application. The time spent grooming other termites was recorded for every individual and then totaled for each replicate. In merged colonies, the direction of grooming (towards nestmates or non-nestmates) was also recorded for each sub-colony within a merged colony (N = 6).

### Survival of single and merged colonies

From the remaining six colonies (colonies O-T), groups of 20 workers were set up with either 20 workers from the same colony (i.e., single colony), or by combining 10 workers each from two different colonies into the same dish (i.e., merged colony; PxQ, PxR, QxR, SxT, SxU and TxU). Groups were isolated in 60 mm petri dishes with moist filter paper and allowed to acclimate for 1 week before exposure. All of these combinations (N = 6) were simultaneously replicated four times with the same pathogen and control solutions. Treatments were applied by pipetting 200 μL of solution onto the filter paper in each petri dish. Mortality was recorded daily for 2 weeks following the solution application.

### Statistical analysis

A Pearson’s χ^2^ test of independence was used to determine if aggressive behavior was associated with pairings between species, within species, or within colonies. Grooming time was compared between single and merged colonies using a nested ANOVA, with colony status (single colony or merged colony) nested within treatment group. We also used a nested ANOVA to determine if grooming in merged colonies was directed more towards nestmates or non-nestmates, with grooming direction nested within treatment group. Using the *coxph* function implemented in the *survival* package in R^107^, termite mortality in the survival assay was analyzed using a Cox proportional hazard survival model with the factor of colony status nested within treatment group. Pairwise comparisons using a log-rank test were performed for the survival distributions of every merged colony and its two corresponding single colonies. To avoid inflation of Type I errors, the Benjamini-Hochberg procedure for adjusting p-values was used. All analyses were performed in the statistical software R 3.5.0^108^.

## Supplementary information

Supplementary Information.

## Data Availability

The data reported in this study have been deposited in the Open Science Framework database, https://osf.io (10.17605/OSF.IO/B73RZ).
