# Supplementary material for: Increased genetic diversity from colony merging in termites does not improve survival against a fungal pathogen
Source: Sci Rep. 2020 Mar 6;10:4212. doi: 10.1038/s41598-020-61278-7 (PMC7060273; doi:10.1038/s41598-020-61278-7)
Supplement: Supplementary file 1 — Supplementary Information. [file 41598_2020_61278_MOESM1_ESM.pdf]

Increased genetic diversity from colony merging in termites does not improve survival against a fungal pathogen

Carlos M. Aguero[1], Pierre-André Eyer & Edward L. Vargo

Department of Entomology, 2143 TAMU, Texas A&M University, College Station, Texas, 77843-2143, USA

[1]Corresponding author:

Carlos Aguero

Department of Entomology,

Texas A&M University,

College Station, 77843, Texas, USA

e-mail: cague001@tamu.edu

Supplementary Information S1

DNA Extraction:

1. Either an individual termite worker or fungal conidia were placed in a 1.5mL tube.
2. 100 µL of cell lysis solution and 1 µL of proteinase K were added to each tube.
3. Samples were ground in solution using a pestle.
4. Samples were placed in a water bath at 55°C for 3 hours.
5. Samples were removed from the water bath and kept in a freezer at -20°C for 30 minutes.
6. 35 µL of 8M ammonium acetate was then added to thawed samples.
7. Samples were centrifuged for 7 minutes at 10K rpm.
8. The supernatant was pipetted into new tubes containing 100 µL cold isopropanol.
9. Samples were then centrifuged for 5 minutes at 10K rpm.
10. Isopropanol was poured out of tubes, leaving behind pelleted DNA.
11. 400 µL 100% Ethanol was added to each sample.
12. Samples were centrifuged for 5 minutes at 10K rpm.
13. Ethanol was poured out, and samples were placed in a vacuum for 15 minutes to remove any excess alcohol.
14. Dry DNA pellets were resuspended in 100 µL of 1X TE buffer overnight.

PCR:

Reaction Template

**Reagent**	**Per Sample**
DNA template	2.0 µL
Forward Primer	0.2 µL
Reverse Primer	0.2 µL
5x PCR Buffer	5 µL
Taq Polymerase	0.06 µL
Water	18.54 µL

Primer sequences and thermocycler programs

<u>Termite - 16S</u>[1]

LR-J-13007 (5'–TTACGCTGTTATCCCTAA-3')

LR-N-13398 (5'-CGCCTGTTTATCAAAAACAT-3')

94°C for 2 min; 41 cycles of 94°C for 45 sec, 50°C for 45 seconds, 72°C for 1 min; final extension 72°C for 5 min.

Fungus – IGS[2]

Ma-IGSspF (5'-CTACCYGGGAGCCCAGGCAAG-3')

Ma-IGSspR (5'-AAGCAGCCTACCCTAAAGC-3')

94°C for 3 min; 30 cycles of 94°C for 1 min, 60°C for 1 min, 72°C for 2 min; final extension 72°C for 5 min.

Sequencing:

Following PCR, amplified DNA was purified using the EXOSAP-it PCR purification kit (Affymetrix), then sequenced using the ABI BigDye Terminator v.3.1 Cycle Sequencing Kit on an ABI 3500 Genetic Analyzer (Applied Biosystems). Base calling and sequence alignment was performed using the software Geneious v.9.1[3]. Aligned sequences were BLAST searched on Genbank for species ID.

References

1 Austin, James W, Allen L Szalanski, Rudolf H Scheffrahn, and Matthew T Messenger. 2005. 'Genetic variation of Reticulitermes flavipes (Isoptera: Rhinotermitidae) in North America applying the mitochondrial rRNA 16S gene', *Annals of the Entomological Society of America*, 98: 980-88.

2 Pantou, Malena P, Annoula Mavridou, and Milton A Typas. 2003. 'IGS sequence variation, group-I introns and the complete nuclear ribosomal DNA of the entomopathogenic fungus Metarhizium: excellent tools for isolate detection and phylogenetic analysis', *Fungal Genetics and Biology*, 38: 159-74.

3 Kearse, Matthew, Richard Moir, Amy Wilson, Steven Stones-Havas, Matthew Cheung, Shane Sturrock, Simon Buxton, Alex Cooper, Sidney Markowitz, and Chris Duran. 2012. 'Geneious Basic: an integrated and extendable desktop software platform for the organization and analysis of sequence data', *Bioinformatics*, 28: 1647-49.

Supplementary Table T1

**Figure 3a**	Control S	Pathogen S	Control S/U	Pathogen S/U	Control U
Pathogen S	2.90E-06	-	-	-	-
Control S/U	0.21272	3.50E-05	-	-	-
Pathogen S/U	1.80E-06	0.55632	1.50E-05	-	-
Control U	0.36613	7.80E-06	0.55632	2.90E-06	-
Pathogen U	4.07E-02	0.00078	0.32749	0.00029	0.14666

**Figure 3b**	Control P	Pathogen P	Control P/R	Pathogen P/R	Control R
Pathogen P	3.90E-12	-	-	-	-
Control P/R	0.5603	1.10E-13	-	-	-
Pathogen P/R	7.00E-12	0.746	2.40E-13	-	-
Control R	0.1602	2.00E-15	0.344	4.40E-15	-
Pathogen R	1.20E-05	0.0021	1.20E-06	0.0031	6.50E-08

**Figure 3c**	Control T	Pathogen T	Control T/U	Pathogen T/U	Control U
Pathogen T	2.35E-03	-	-	-	-
Control T/U	0.17454	5.30E-04	-	-	-
Pathogen T/U	2.35E-03	0.75188	5.30E-04	-	-
Control U	0.38616	4.60E-04	0.4414	4.60E-04	-
Pathogen U	5.09E-01	0.01411	8.23E-02	0.01413	1.47E-01

**Figure 3d**	Control Q	Pathogen Q	Control Q/R	Pathogen Q/R	Control R
Pathogen Q	1.50E-02	-	-	-	-
Control Q/R	0.111	3.16E-01	-	-	-
Pathogen Q/R	1.50E-06	0.013	6.20E-05	-	-
Control R	1	1.10E-02	0.095	5.10E-07	-
Pathogen R	9.70E-07	0.011	3.70E-05	0.983	4.50E-07

**Figure 3e**	Control S	Pathogen S	Control S/T	Pathogen S/T	Control T
Pathogen S	6.50E-12	-	-	-	-
Control S/T	0.99	6.50E-12	-	-	-
Pathogen S/T	2.74E-01	1.70E-07	2.74E-01	-	-
Control T	0.212	1.00E-13	0.212	4.00E-02	-
Pathogen T	2.12E-01	9.50E-07	2.12E-01	8.86E-01	2.40E-02

**Figure 3f**	Control P	Pathogen P	Control P/Q	Pathogen P/Q	Control Q
Pathogen P	1.40E-06	-	-	-	-
Control P/Q	1	1.40E-06	-	-	-
Pathogen P/Q	9.20E-09	1.27E-01	9.20E-09	-	-
Control Q	0.1017	7.20E-05	0.1017	5.90E-07	-
Pathogen Q	2.50E-05	6.26E-01	2.50E-05	8.92E-02	1.50E-03

Pairwise comparisons of survival distributions using a Log-Rank test. Each table corresponds to one of the plots of survival curves in Figure 3. All analyses were performed in the statistical software R 3.5.0 (https://www.r-project.org/).
